# Trilogy of Fallot With a Large Right Atrial Thrombus: A Case Report

**DOI:** 10.7759/cureus.79395

**Published:** 2025-02-21

**Authors:** Ramachandra Barik, Debasis Acharya, Pranjit Deb, Debasis Panda, Sindhu Rao Malla

**Affiliations:** 1 Cardiology, All India Institute of Medical Sciences, Bhubaneswar, Bhubaneswar, IND

**Keywords:** balloon valvotomy, cyanosis, paradoxical embolism, pulmonary stenosis, thrombus, trilogy of fallot

## Abstract

Trilogy of Fallot is a rare congenital heart disease consisting of pulmonary stenosis, right-to-left interatrial shunt, and right ventricular hypertrophy. It can lead to complications such as thrombus formation as a result of chronic hypoxia leading to erythrocytosis and hyperviscosity, and paradoxical embolisms due to the right-to-left interatrial shunt. This report illustrates a case of a young man who presented with cyanosis and right heart failure with a history of recurrent episodes of transient ischemic attacks. He was found to have Trilogy of Fallot with a large right atrial thrombus. He showed significant improvement following treatment with low molecular weight heparin and thereafter undergoing balloon pulmonary valvotomy.

## Introduction

Pulmonary stenosis (PS) is a rare condition, affecting roughly 1 in every 2000 live births globally, and constitutes around 8% of all cases of congenital heart disease (CHD) [1]. It is typically congenital in origin [2]. It is most commonly associated with a ventricular septal defect, dextroposed aorta, and right ventricular (RV) hypertrophy (Tetralogy of Fallot). However, it can also present with an intact ventricular septum wherein the majority of the cases also have a patent foramen ovale [3]. The triad of PS, an interatrial shunt, and RV hypertrophy is known as the Trilogy of Fallot. This combination of defects can lead to thrombus formation and recurrent paradoxical embolisms due to the presence of a right-to-left interatrial shunt. We present a case of a young gentleman who had multiple transient ischemic attacks secondary to the Trilogy of Fallot and was successfully managed with anticoagulation and balloon pulmonary valvotomy.

## Case presentation

A 26-year-old man presented with a history of exercise intolerance and dyspnoea of New York Heart Association (NYHA) class II for 12 years, which was insidious in onset. The dyspnoea gradually worsened to NYHA class IV in the past two years; however, the patient could not report any specific triggering factors. He noticed bluish discoloration in his tongue and lips for the last five months, which was sudden in onset. He also noticed swelling in both feet for the last one month associated with abdominal distension. Additionally, he reported multiple episodes of weakness in his right upper and lower limbs associated with slurring of speech which lasted for 30 minutes and resolved spontaneously. The patient had visited several hospitals for dyspnoea on exertion where he was prescribed diuretics; however, no investigations were done other than routine blood investigations. CHD screening was not performed. There is no history of surgery or intervention in the past.

On physical examination, BMI was 15.8 kg/m^2^. He had central cyanosis, severe clubbing of fingers and toes suggestive of long-standing hypoxia, and bilateral pitting pedal edema. He was found to have a blood pressure of 120/80 mmHg, heart rate of 80 beats/min, respiratory rate of 34 breaths/min, and oxygen saturation of 70% in room air. Cardiovascular examination revealed parasternal heave and diminished P2 component of the second heart sound. There was a Levine grade IV/VI ejection systolic murmur in the second intercostal space at the left sternal border radiating to the left shoulder which started with a click. There were no additional heart sounds. Neurological examination was normal.

**Figure 1:**
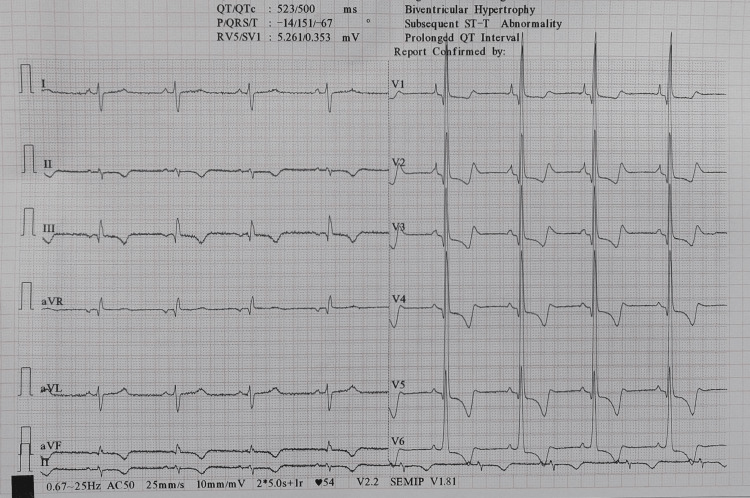
ECG showing right axis deviation, right atrial enlargement, and right ventricular hypertrophy with a strain pattern.

Routine investigations showed a hemoglobin of 18.1 gm/dL indicating polycythemia. ECG showed right axis deviation, right atrial abnormality, and RV hypertrophy with strain pattern (Figure 1).

**Figure 2:**
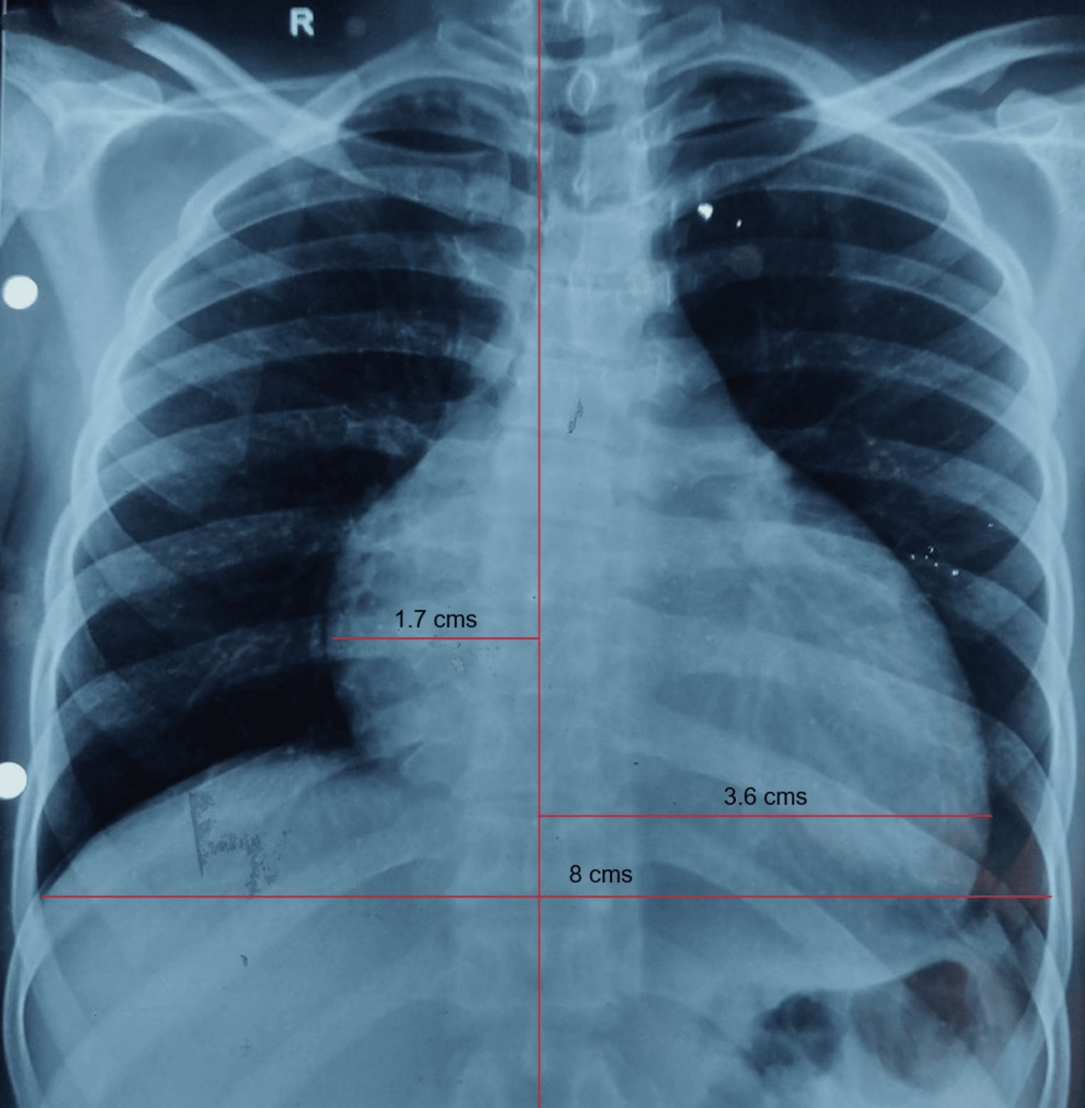
Chest X-ray (CXR) showing cardiomegaly with a cardiothoracic ratio of 0.66 and oligemic lung fields.

Chest X-ray showed cardiomegaly and oligemic lung fields (Figure 2).

**Figure 3:**
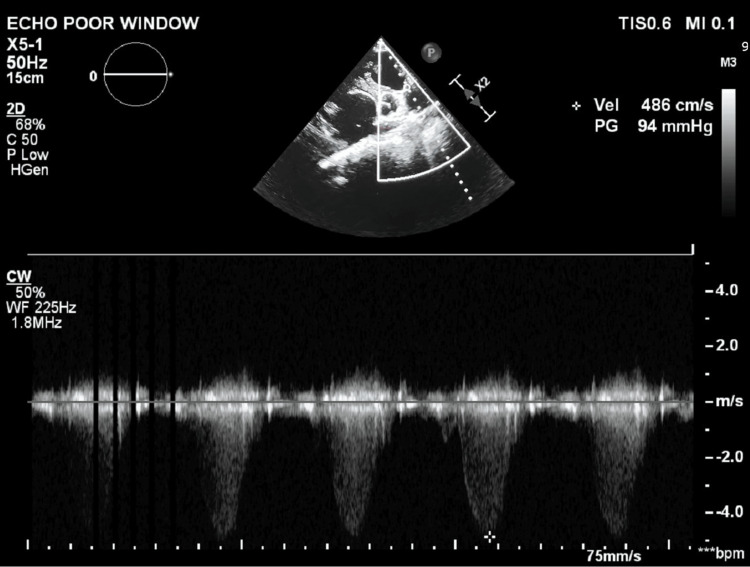
Transthoracic echocardiography (ECHO) in the parasternal short-axis view with continuous wave Doppler across the pulmonary valve, demonstrating severe valvular pulmonary stenosis with a pressure gradient of 94 mmHg.

**Figure 4:**
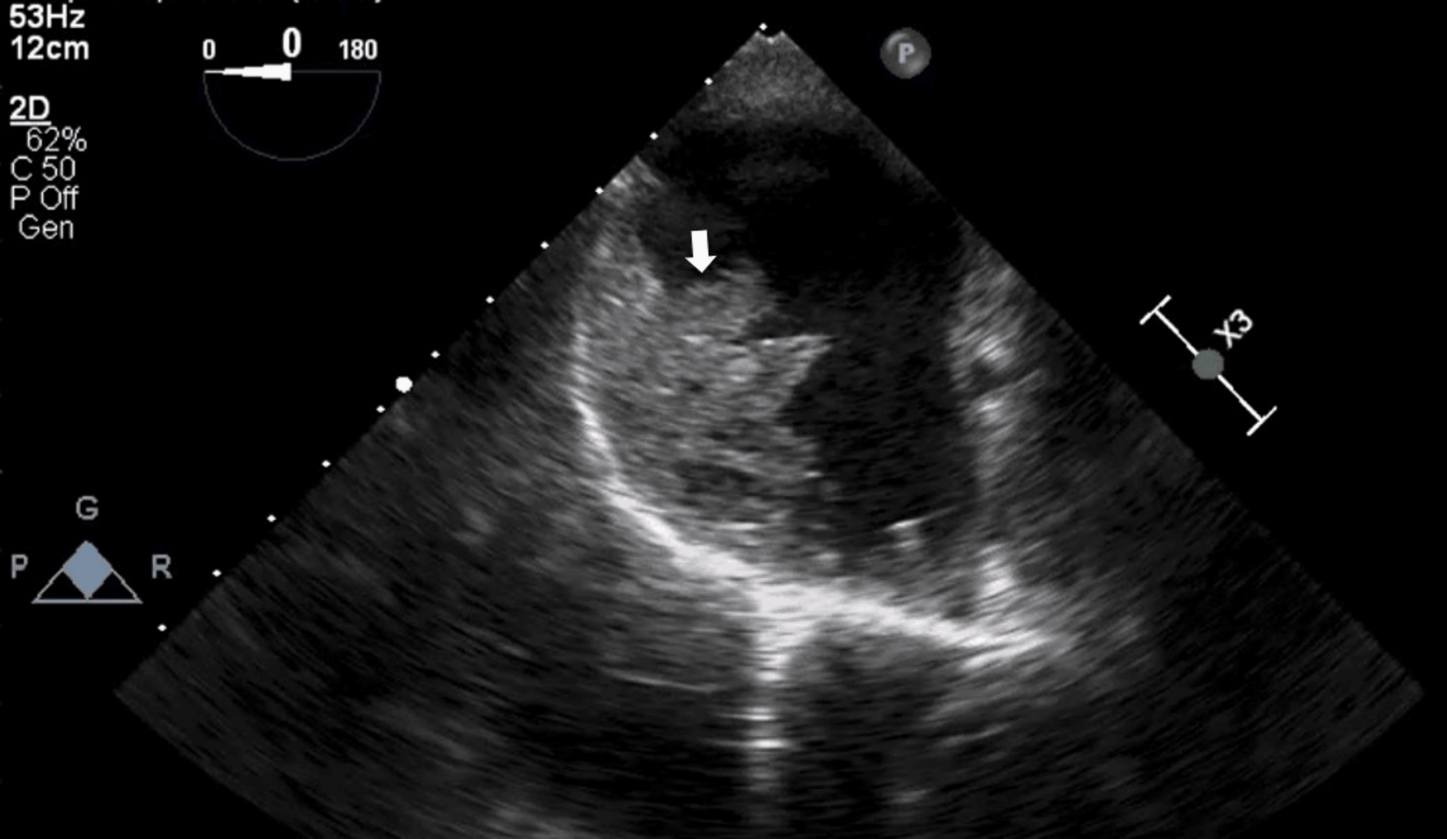
Transesophageal echocardiography (ECHO) showing a large right atrial thrombus (white arrow) measuring 8.7 × 12.1 cm.

Transthoracic echocardiography (ECHO) revealed a large well-defined echogenic mass in the right atrium measuring 8.7 x 12.1 cm in size. It also showed dilated right atrium and right ventricle with moderate tricuspid regurgitation and normal biventricular function. There was a domed appearance of the pulmonary valve with severe stenosis and a gradient of 97 mmHg (Figure 3) with an RV systolic pressure gradient of 120 mmHg and post-stenotic dilatation of the pulmonary trunk. Transesophageal ECHO showed the same (Figure 4) and revealed a patent foramen ovale with a right to left shunt confirmed by an agitated saline contrast study. Computed tomography pulmonary angiography (CTPA) confirmed that there were no abnormal pulmonary or systemic venous drainage anomalies.

The patient was initiated on low molecular weight heparin in view of suspicion of right atrial thrombus and was closely monitored for any clinical deterioration. Surgical options were not initially considered, as complete resolution with anticoagulation alone was anticipated. Repeat ECHO after 15 days showed no residual thrombus fragments.

**Figure 5:**
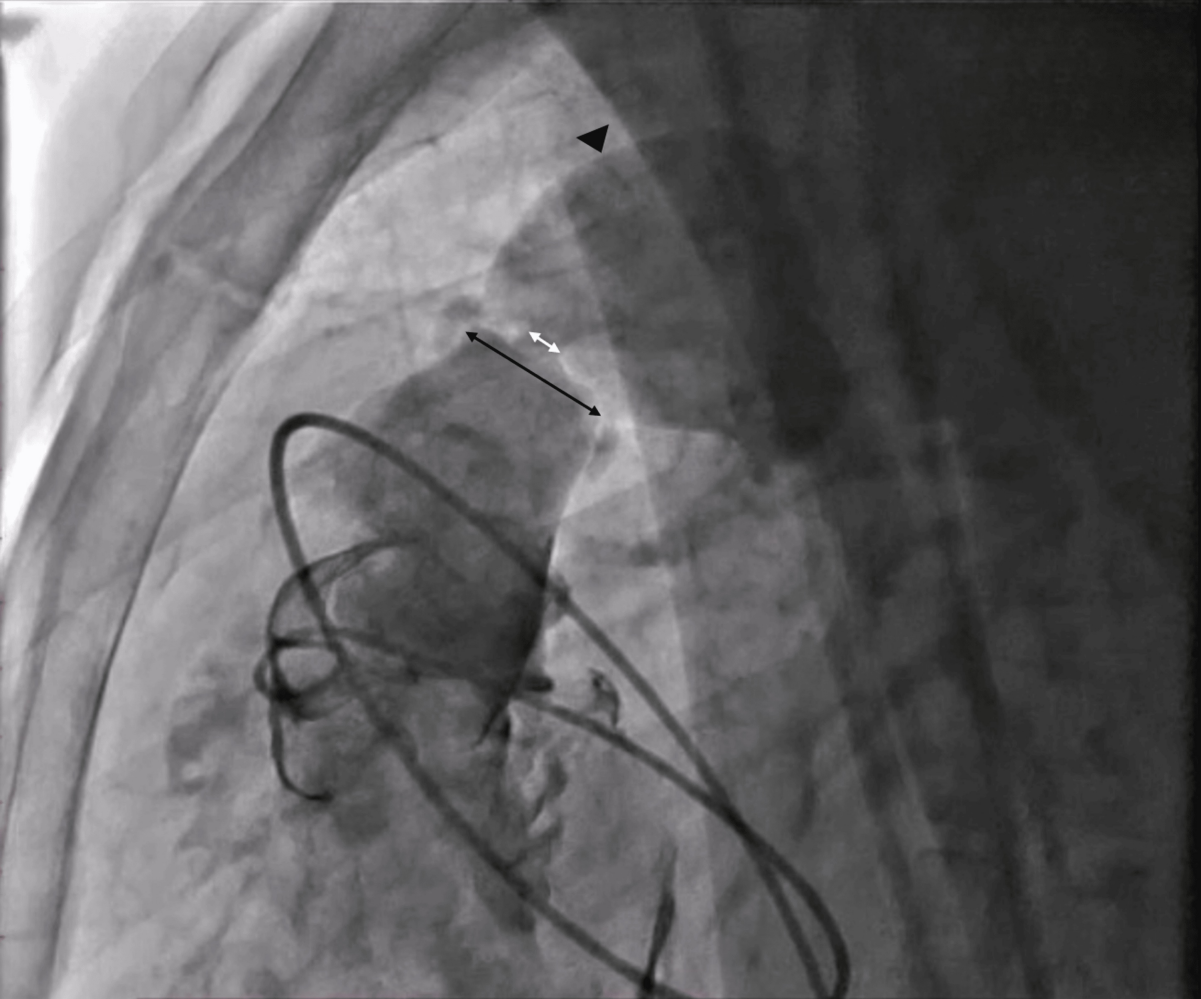
Right ventricular (RV) angiogram in the left lateral view showing the stenotic orifice (white line arrow) and the pulmonary valve annulus measuring 26 mm (black line arrow) with post-stenotic dilatation (black arrowhead).

Subsequently, the patient was taken up for percutaneous balloon pulmonary valvotomy. Through right femoral vein access, under fluoroscopic guidance, a 5F pigtail catheter was advanced into the right ventricle. The RV systolic pressure was measured at 200 mmHg and pulmonary artery (PA) systolic pressure at 20 mmHg (pulmonary transvalvular gradient - 180 mmHg) indicating severe PS. RV angiogram was taken in the left lateral view to visualize the orifice and measure the annulus of the pulmonary valve which was 26 mm (Figure 5).

**Figure 6:**
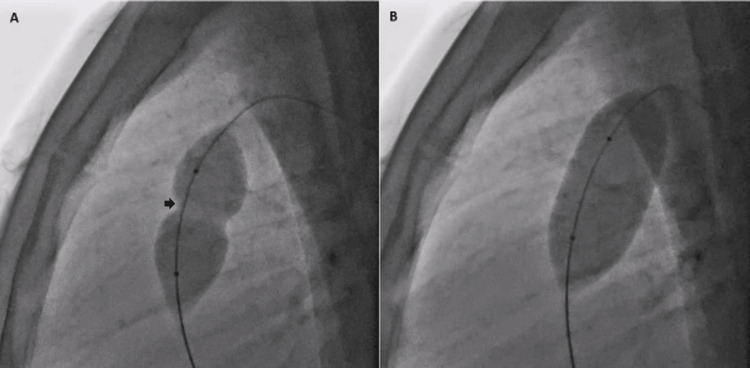
Fluoroscopy in the left lateral view showing inflation of the Tyshak balloon positioned across the pulmonary valve. (A) Initially, a waist (black arrow) is seen at the level of the valve due to severe stenosis. (B) The waist disappears with further inflation of the balloon, indicating successful dilatation of the pulmonary valve.

Then a Tyshak balloon (NuMED Inc., New York, USA) of size 22 mm x 30 mm was positioned across the pulmonary valve and inflated and deflated repeatedly (Figure 6).

Invasive pressure measurements post-dilatation revealed RV systolic pressure to be 80 mmHg and PA systolic pressure to be 52 mmHg. The pulmonary transvalvular pressure gradient dropped significantly to 28 mm Hg and remained stable (Table 1). The patient's saturation improved to 98% on room air on the cath lab table. There were no periprocedural complications.

**Table 1:** Table summarizing hemodynamic parameters before and after balloon pulmonary valvotomy. RV: right ventricular; PA: pulmonary artery

Parameter	Pre-valvotomy	Post-valvotomy
RV systolic pressure (mmHg)	200	80
PA systolic pressure (mmHg)	20	52
Pulmonary valve gradient (mmHg)	180	28
Oxygen saturation (mmHg)	70	98

**Figure 7:**
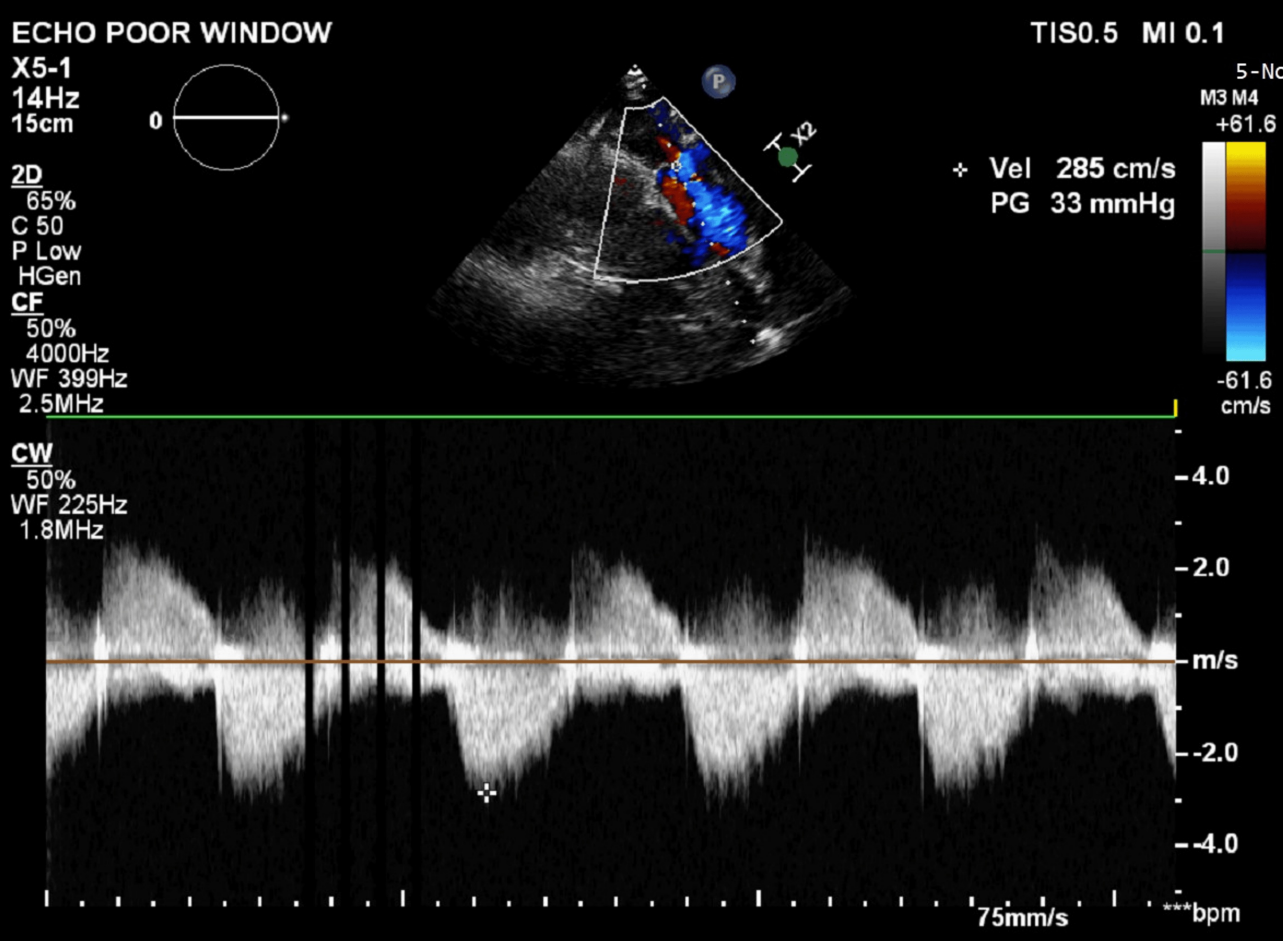
Follow-up echocardiography (PLAX RV outflow view) showing a pulmonary valve gradient of 33 mmHg with mild pulmonary regurgitation. PLAX: parasternal long-axis; RV: right ventricular

The patient was discharged in a stable condition with low-dose aspirin, vitamin K antagonist (acenocoumarol), and diuretics. On follow-up, the patient reported significant improvement in exercise tolerance, now in NYHA class I. On examination, there was no pedal edema or cyanosis, and oxygen saturation was normal. ECHO showed no clot in the right atrium and a pulmonary valve gradient of 32 mm Hg with mild pulmonary regurgitation (Figure 7). It also showed a reduction in right atrial and ventricular dimensions. Hypercoagulability testing was not performed as there was a clear identifiable trigger which was treated and no recurrence of thrombus even after three months of follow-up. Anticoagulation and aspirin were stopped after three months.

## Discussion

The triad of PS with the intact ventricular septum, RV hypertrophy, and a defect in the atrial septum was first described by Fallot in seven patients in 1888; however, he did not explicitly differentiate it from Tetralogy of Fallot [4]. Its prevalence ranges between 1.2% and 6% of CHD [5]. This rare condition can be broadly categorized into two distinct subtypes. The first subtype is mild to moderate PS and a large atrial septal defect. This is characterized by a left-to-right shunt at the atrial level, leading to increased pulmonary blood flow, and eventually RV volume overload and failure if left untreated. The second subtype is severe PS with a right-to-left interatrial shunt, usually occurring through a patent foramen ovale. This is associated with decreased pulmonary blood flow and the right to left shunting in this subtype leads to chronic hypoxemia and cyanosis [6].

The persistence of the interatrial shunt and PS has adverse consequences on the right ventricle and the individual's exercise capacity gradually declines [7]. The right ventricle initially undergoes concentric hypertrophy in response to the elevated wall stress due to chronic pressure overload. Gradually, this adaptation becomes insufficient to counteract the increased afterload; hence, the right ventricle eventually undergoes maladaptive remodeling. This results in eccentric hypertrophy, dilatation, impaired systolic and diastolic function, and ultimately RV failure [8].

PS with a patent foramen ovale is marked by chronic cyanosis, polycythemia, and clubbing. It falls in between the Tetralogy of Fallot and Eisenmenger syndrome in terms of the severity of cyanosis [9]. Cyanotic patients are particularly vulnerable to paradoxical embolisms due to a persistent right-to-left interatrial shunt. They have a 10-fold higher risk of stroke compared to acyanotic congenital heart defects due to increased thrombotic risk [10]. Thrombus formation can occur due to raised hematocrit, stasis of blood within dilated chambers and vessels, coagulation abnormalities, atrial arrhythmias, endothelial dysfunction, atherosclerotic changes, infective endocarditis, and pregnancy [11]. Despite this, current data do not support routine anticoagulation in cyanotic patients, unless associated with atrial arrhythmias, prior thromboembolic events, or a history of intracardiac or intravascular thrombi [10].

There are very few case reports on the Trilogy of Fallot and our case is unique in that the patient presented with a large right atrial thrombus and a history of recurrent transient ischemic attacks. To the best of our knowledge, this is the first case to be reported in the literature. Early echocardiographic screening in cyanotic patients with neurological symptoms can aid in identifying intracardiac shunts and thrombi, thus influencing decisions regarding anticoagulation and surgical or transcatheter interventions. Balloon pulmonary valvotomy is the first line of treatment for pulmonary valvar stenosis and should be performed promptly upon diagnosis. It has been shown to immediately reduce pressure gradients and increase RV outflow. Additionally, it leads to improvements in RV function, tricuspid regurgitation, and right-to-left shunting [12].

## Conclusions

Trilogy of Fallot is a rare congenital heart defect that can cause erythrocytosis and an increase in blood viscosity due to chronic hypoxemia. This combined with local stasis secondary to right atrial enlargement can lead to thrombus formation and paradoxical embolism. The patient had immediate improvement in symptoms and oxygen saturation following balloon pulmonary valvotomy, demonstrating the importance of early diagnosis and intervention. Prompt recognition and appropriate management are critical in patients with CHD to prevent complications and improve outcomes.
